# Graphene Functionalization by O_2_, H_2_, and Ar Plasma Treatments for Improved NH_3_ Gas Sensing

**DOI:** 10.1021/acsami.4c17257

**Published:** 2024-12-26

**Authors:** Sogo Iwakami, Shunya Yakushiji, Tomonori Ohba

**Affiliations:** Graduate School of Science, Chiba University, 1-33 Yayoi, Inage, Chiba 263-8522, Japan

**Keywords:** graphene, graphane, oxidized graphene, plasma treatment, NH_3_ gas sensor

## Abstract

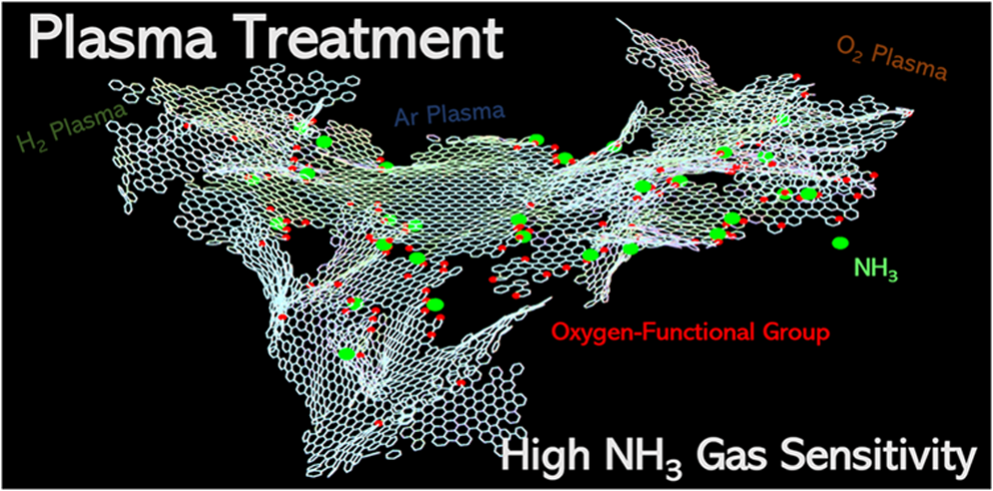

Graphene-based materials have gained attention for their promise in various applications owing to their two-dimensional structure. Functionalizing the graphene surface can help realize materials with noble properties. In this study, graphene was functionalized by plasma treatment in O_2_, H_2_, and Ar environments, and the effects on the NH_3_ gas-sensing performance were evaluated. The O_2_ plasma treatment induced oxidation of the graphene (i.e., graphoxide), while the H_2_ plasma treatment induced hydrogenation (i.e., graphane). Raman scattering spectroscopy suggested that graphoxide had vacancy-type defects and graphane had sp^3^-type defects, while Ar-treated graphene had both types of defects. Graphane had the highest sheet resistance followed by graphoxide, Ar-treated graphene, and pristine graphene, which can be attributed to the large bandgap of 3.0 eV for graphane. In contrast, graphoxide had the best NH_3_ gas-sensing performance, which indicates that NH_3_ gas interacts more strongly with vacancy-type defects than with sp^3^-type defects. The results showed that functionalizing the graphene structure generated noble materials with a superior NH_3_ gas-sensing performance compared with pristine graphene.

## 1 Introduction

Gas sensing technology is important for monitoring pollutant gases. Solid-state gas sensors detect a target gas by a catalytic reaction.^1−3^ New gas sensors have been developed that use carbon nanomaterials such as carbon nanotubes^4^ and graphene.^5^ Graphene is a representative two-dimensional carbon nanomaterial with superior properties such as high electrical conductivity,^6−8^ chemical stability, and mechanical strength^9−11^ that make it an attractive candidate material for gas sensors and optically transparent conductors. Graphene-based gas sensors have demonstrated high adsorption sensitivity and selectivity in which the electrical conductivity changes according to the local carrier concentration at adsorption sites. The sheet resistance was thus changed positively or negatively when the target gas is adsorbed.^12,13^ Metal-doping on graphene has been also shown to improve the gas sensing performance considerably.^14−17^

The adsorption energy between a gas molecule and graphene can be increased by introducing defects on the graphene surface. Mattson and co-workers demonstrated that the NH_3_ adsorption energy is higher at a carbon vacancy site (−1.71 eV) than on pristine graphene (−0.12 eV).^18^ Peng and co-workers demonstrated that the NH_3_ adsorption energy is higher for the epoxy group (0.14 eV) and hydroxy group (0.53 eV) than on pristine graphene (0.10 eV).^19^ Reduced graphene oxides have high gas sensitivity because of their smooth surfaces and defects, which increase their electrical conductivity and number of active sites, respectively.^20−22^ Minitha and co-workers showed that reduced graphene oxides with a large amount of oxygen functional groups demonstrated a high gas-sensing response, which however had poor recovery (i.e., incomplete desorption of the adsorbed gas).^20^ In contrast, reduced graphene oxides with fewer oxygen functional groups demonstrated a moderate gas-sensing response with complete recovery. Yang and co-workers etched graphene oxide in a photo-Fenton reaction to prepare a reduced holey graphene oxide that demonstrated an excellent NH_3_ sensing performance with a high responsivity, fast response, and short recovery time.^21^ Edge sites on graphene also work as active adsorption sites; Huang and co-workers used first-principle calculations to show that the armchair edges on graphene nanoribbons have high sensitivity to NH_3_ gas but are insensitive to CO, NO, NO_2_, O_2_, N_2_, and CO_2_.^23^ The high NH_3_ sensitivity of graphene nanoribbons and nanocrystalline graphene has been attributed to chemisorption.^23,24^

Functionalizing graphene can thus greatly improve the gas-sensing performance. However, developing a fundamental understanding of how functional groups on graphene control the gas-sensing performance is necessary. Our preceding study on gas sensing on nanocrystalline graphene indicated that NH_3_ had the largest resistance change in NH_3_, H_2_, CO_2_, and He gases.^21^ Therefore, in this study, graphene was functionalized by different plasma treatments, and the effects on the NH_3_ gas adsorption mechanism and electrical conductivity were evaluated.

## 2 Materials and Methods

Graphene was synthesized as follows (Figure S1).^25^ Cu foil (18 mm × 18 mm × 0.08 mm, CU-113303, Nilaco, Tokyo, Japan) was heated to 1300 K and then annealed for 30 min in a mixture of H_2_ as the reductive gas (20 cm^3^ min^–1^) and Ar as the flow gas (500 cm^3^ min^–1^). Under the above temperature and gas flow conditions, graphene was synthesized on the Cu substrate by chemical vapor deposition using CH_4_ at a flow rate of 2.0 cm^3^ min^–1^. The CH_4_ flow was then stopped, and the graphene on Cu substrate was cooled to room temperature using the flow of mixed H_2_ and Ar gases. The graphene on Cu substrate was coated by polycarbonate (PC) in a chloroform solution (200 g L^–1^) and was then heated to 473 K for 10 min in a vacuum. One side of the graphene was scratched to expose the Cu substrate, which was then etched by a 0.1 mol L^–1^ (NH_4_)_2_S_2_O_8_ solution. The graphene/PC was placed on a quartz substrate (20 mm × 20 mm × 1.0 mm, Kenis, Osaka, Japan), washed in distilled water for 2 h to remove residual Cu ions, and dried in air for over 4 h. Graphene was functionalized by plasma treatment with O_2_, H_2_, or Ar gas at a pressure of 25 Pa and current of 30 mA for 5, 10, 15, or 20 s (PIB-20, Vacuum Device Co., Ibaraki, Japan). The O_2_ plasma treatment resulted in oxidation of the graphene (i.e., graphoxide) while the H_2_ plasma treatment resulted in hydrogenation of the graphene (i.e., graphane).^26^

Raman scattering spectra were obtained by laser irradiation at a wavelength of 532 nm (NRS-3100, JASCO Co., Tokyo, Japan) and X-ray photoelectron spectroscopy (XPS) was performed using a Mg Kα radiation source (JPS-9030, JEOL Co., Tokyo, Japan) to determine the chemical structures of the functionalized graphene. The XPS spectra were averaged by using three-to-five different graphene samples with the same plasma treatment for 20 s and two-to-three different graphene samples with those for 5 s. The graphene layer numbers were determined using ultraviolet (UV)–visible (VIS) spectroscopies (UV-2600i, Shmazu Co., kyoto, Japan). The four-probe method was performed by using a DC voltage/current generator (ADCMT, 6146, ADC Co., Saitama, Japan; Picoscope5203, Pico Technology Co., Cambridgeshire, United Kingdom) at a current of 0.1–1.5 mA to determine the electrical resistance (Figure S2). The sheet resistance (*R*_S_) is defined as *R*_S_ = *R*·*W*/*L*, where *R*, *W*, and *L* are the electrical resistance, width of an electrode, and length between electrodes, respectively. Pristine graphene and the different types of functionalized graphene were preheated to 360 K for 3 h in a vacuum, and their NH_3_ gas-sensing performances were then examined at 273 K in a static system. The bandgap of pristine graphene was evaluated from the sheet resistances at 113, 203, 280, and 370 K under the relationship between the resistance and temperature; ln(*R*_1_/*R*_2_) = *E*_g_/2*k*_B_ (1/*T*_1_ – 1/*T*_2_) where *R*, *E*_g_, *k*_B_, and *T* are the sheet resistance, the bandgap energy, Boltzmann constant, and temperature. NH_3_ gas was supplied at pressures of 0.1, 0.3, 1.0, and 3.2 kPa after evacuation using vacuum pump. Density functional theory (DFT) calculations were performed using the Vienna ab initio simulation package (VASP)^27^ to obtain the electronic structures. All calculations were conducted based on the projector augment wave pseudopotentials and Perdew–Burke–Ernzerhof exchange correlation function.^28^ The Brillouin zone was sampled by using a 16 × 16 × 8 mesh in the Monkhorst–Pack scheme.^29^

## 3 Results and Discussion

### 3.1 Chemical Structure

**Figure 1:**
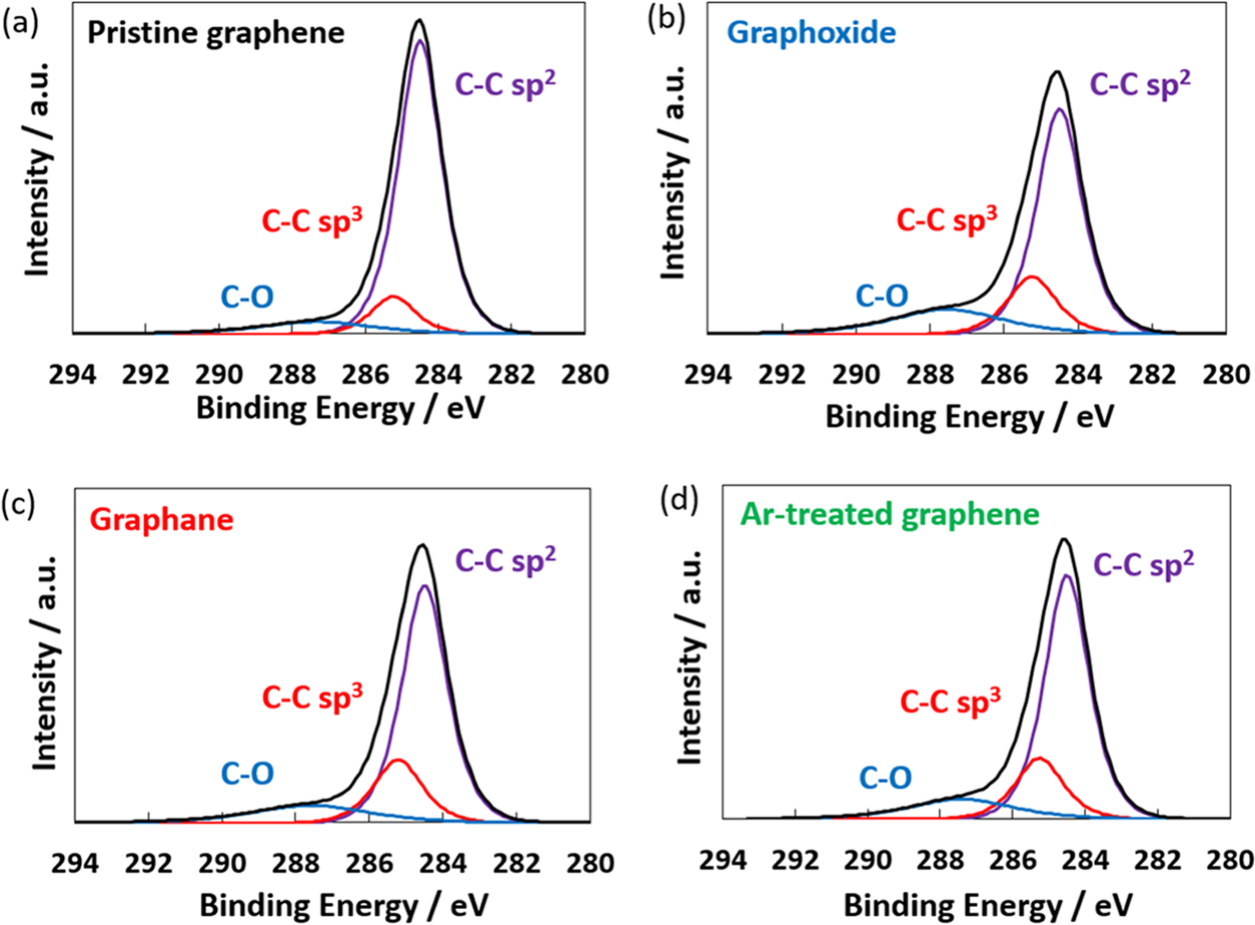
XPS C 1s spectra of (a) pristine graphene, (b) graphoxide, (c) graphane, and (d) Ar-treated graphene at the treatment time of 20 s.

Figure S3 shows the UV–vis spectra of graphene samples to determine graphene layer numbers for the pristine graphene and the plasma-treated graphene for 20 s. The transmittances of graphene, graphoxide, graphane, and Ar-treated-graphene were 88.9 ± 0.9, 91.8 ± 0.1, 91.1 ± 0.3, and 92.4 ± 0.7%, respectively. The graphene layer numbers were thus determined to be 4.3 ± 0.4, 3.1 ± 0.1, 3.4 ± 0.1, and 2.9 ± 0.3 in the above order, based on the absorption of 2.6% per graphene layer at 550 nm.^30,31^ Approximately one layer of graphene was thus disengaged by the plasma treatment. Figure 1 shows the XPS C 1s spectra of the pristine graphene and different types of functionalized graphene treated for 20 s (Figure S4 shows the XPS C 1s spectra of the different types of functionalized graphene treated for 5 s). The C 1s peaks at 284.5, 285.2, and 287.5 eV corresponded to sp^2^ hybridized bonds, sp^3^ hybridized bonds, and oxygen functional groups, respectively. The oxygen functional groups were attributed to C–OH at 286.4 eV, C–O–C at 287 eV, and C=O at 288 eV, whereas a C–OOH group at 289 eV was hardly observed.^32^ The C–O–C and C=O groups were thus predominantly introduced by the plasma treatment. The areas of the peaks were used to determine the atomic ratios for the pristine and functionalized graphene, as listed in Table 1. Pristine graphene mainly comprised sp^2^ carbon (81%). The functionalized graphene treated for 5 s showed decreases in sp^2^ carbon (75–78%) and increases in sp^3^ carbon and oxygen functional groups (Table S1). The functionalized graphene treated for 20 s showed larger decreases in sp^2^ carbon (65–68%) and increases in sp^3^ carbon and oxygen functional groups. Graphoxide had the largest amount of oxygen functional groups (17%) owing to the oxygenation of the basal planes and/or edges of graphene by the reactive O_2_ plasma. Figure S5 shows the ratio between the oxygen functional groups and sp^3^ carbon for the pristine and functionalized graphene. Graphane and Ar-treated graphene were expected to have more sp^3^ carbon and fewer oxygen functional groups than pristine graphene, because the H_2_ and Ar plasma treatments could create sp^3^-type defects and remove oxygen functional groups. Although graphane and Ar-treated graphene showed increases in sp^3^ carbon, they also had more oxygen functional groups (13%) than pristine graphene (8.7%). However, those graphene had fewer oxygen functional groups, compared with graphoxide. The increase in oxygen functional groups may be because the plasma treatment created defective structures including sp^3^ carbons with dangling bonds, which were then oxidized by O_2_ and water vapor after exposure to air.

**Table 1:** Ratios of sp^2^ Carbon, sp^3^ Carbon, and Oxygen Functional Groups for Pristine and Functionalized Graphene^a^

	sp^2^ carbon	sp^3^ carbon	oxygen functional groups
pristine graphene	80.9% ± 1.3%	10.4% ± 1.4%	8.7% ± 0.2%
graphoxide	65.0% ± 3.8%	17.6% ± 2.1%	17.4% ± 2.6%
graphane	68.3% ± 4.2%	18.4% ± 2.1%	13.2% ± 2.5%
Ar-treated graphene	67.8% ± 4.4%	19.1% ± 2.4%	13.1% ± 2.3%

a Note: Functionalized graphene types were treated for 20 s. Values were obtained by calculating the areas under the peaks in the XPS C 1s spectra.

**Figure 2:**
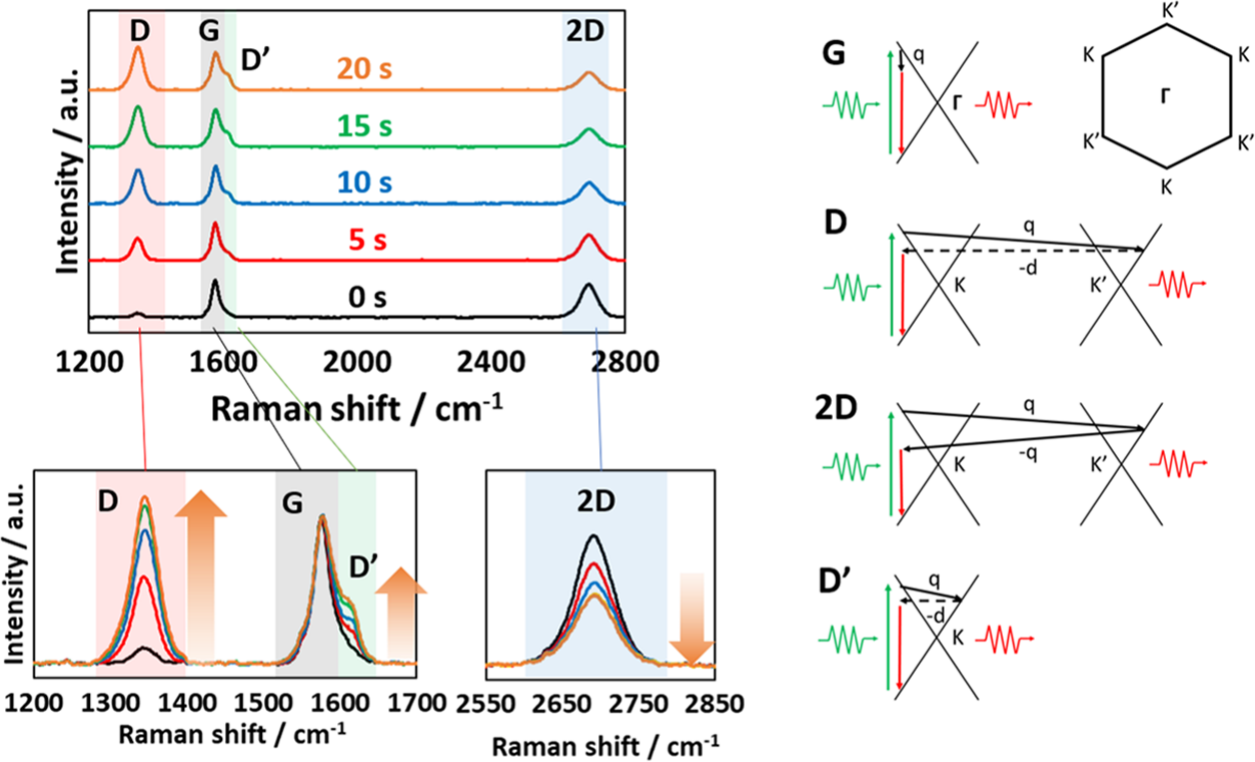
Raman scattering spectra of Ar-treated graphene according to treatment time.

Figure 2 shows the changes in Raman spectra of Ar-treated graphene with treatment times of 5, 10, 15, and 20 s (see also Figure S6 for graphane and graphoxide). Those spectra were here normalized by the intensities of the G-band. The G band at ∼1580 cm^–1^ was attributed to a doubly degenerate in-plane sp^2^ C–C stretching mode belonging to the *E*_2*g*_ irreducible representation.^32^ The 2D band at ∼2700 cm^–1^ was attributed to a second-order Raman process originating from the in-plane breathing-like mode of the carbon rings belonging to the totally symmetric irreducible representation *A*_1_′ at the *K* or *K*′ point in the first Brillouin zone. The electron was inelastically scattered by an iTO phonon between the *K* and *K*′ points. Similar to the 2D band, the D band at ∼1350 cm^–1^ was attributed to an iTO phonon around the *K* point, but the electron was inelastically scattered to the *K*′ point and was then elastically backscattered to the *K* point by a defect.^33^ The D′ band at ∼1620 cm^–1^ was attributed to an intravalley process near the *K* point, which requires a defect.^34^ The D and D′ bands increased relative to the G band by Ar plasma treatment while the 2D band decreased. The same trends were observed for graphoxide and graphane.

The D band is generally considered to be related to disorder/defects on the graphene structure through a disorder-induced double resonance Raman process. The D′ band has also been attributed to a disorder-induced double resonance Raman process that is sometimes observed in nanocrystalline graphite.^35^ Thus, the increases in the D and D′ bands indicate that defects were inserted on graphene and that the average size of crystalline graphene units decreased. Although the 2D band is often linked to the number of graphene layers (i.e., a smaller 2D band indicates more graphene layers),^36^ it is an overtone of the D band, and the D and 2D bands compete each other. As the plasma treatment could not increase the number of graphene layers and actually the layer number was slightly decreased by the plasma treatment shown in Figure S3, the decrease of 2D/D band ratio is a result of the increased D band intensity as well as the decrease in the 2D band intensity.

**Figure 3:**
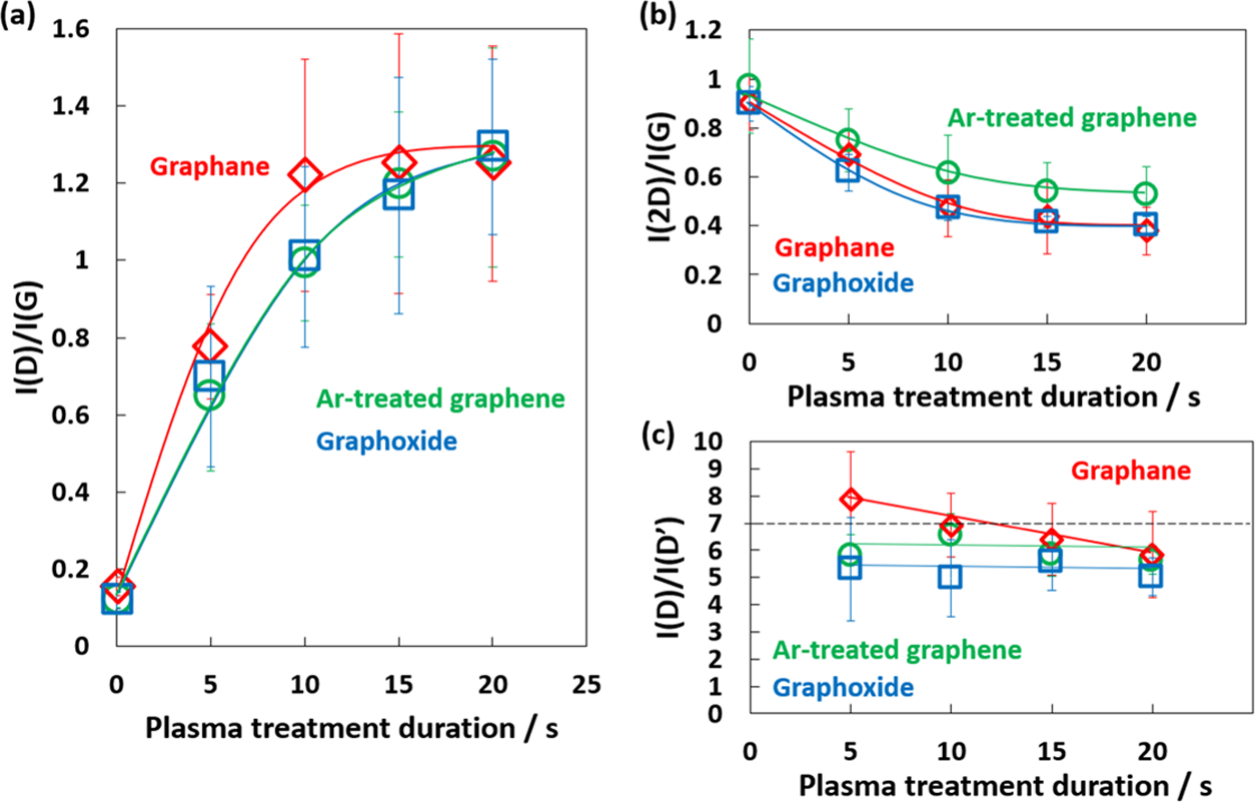
Changes in Raman scattering spectra with treatment time for graphoxide (blue), graphane (red), and Ar-treated graphene (green): (a) *I*(D)/*I*(G), (b) *I*(2D)/*I*(G), and (c) *I*(D)/*I*(D′).

Figure 3 shows the changes in intensity ratios of D/G, 2D/G, and D/D′ with treatment time of the different types of functionalized graphene. Although the trends were similar, some differences were observed among the different types of functionalized graphene. Graphane showed a quicker increase in the D band intensity than the others, which indicates that hydrogenation promoted the transformation from graphene to disordered graphane structures. Davydova and co-workers conducted molecular dynamics simulations to investigate the damage to graphene induced by H^+^ ions and showed that low-energy H^+^ ion exposure fully hydrogenated graphene before graphene vacancies were created.^37^ Graphoxide and graphane showed similar large decreases in intensity of the 2D band compared with Ar-treated graphene. Meanwhile, graphoxide and Ar-treated graphene showed similar changes in the D band intensity. These results suggest that the O_2_ plasma treatment greatly influenced the 2D band. Das and co-workers measured the changes in Raman spectra of graphene with electrochemical top gating and proposed that hole or electron doping decreased the 2D band intensity.^38^ Thus, the oxygen functional groups inserted by O_2_ plasma treatment may have taken an electron from graphene, which would decrease the 2D band intensity. The mechanism on the electrostatic attraction of an electron to oxygen atoms was supported from the DFT calculation, as shown in Figure S7.

The plasma treatments increased the D′ band (Figures 2 and S6), while pristine graphene had no D′ band. The *I*(D)/*I*(D′) ratio is widely used to estimate the kind of defects in graphene. Casiraghi, Zandiatashbar, and Zhao measured the changes in the Raman spectra of graphene with the introduction of different types of defects and concluded that *I*(D)/*I*(D′) > 7 indicates sp^3^-type defects are dominant while *I*(D)/*I*(D′) < 7 indicates that vacancy-type defects are dominant. *I*(D)/*I*(D′) ≃ 5 for graphoxide, which indicates that oxidation predominantly led to vacancy-type defects.^39−41^ Pei and co-workers used molecular dynamics simulations to propose the following defect formation scheme for O_2_ plasma treatment:^41^ Chemisorption of oxygen on carbon forms sp^3^- and vacancy-type defects, which is followed by the rapid destruction of the graphene structure by carbon removal. *I*(D)/*I*(D′) ≃ 8 for graphane with a treatment time of 5 s, which decreased to *I*(D)/*I*(D′) ≃ 6 with increasing treatment time, which indicates that sp^3^-type defects were predominant. Thus, the hydrogenation of graphene by H_2_ plasma treatment induced the formation of sp^3^ carbons with less vacancy defects, which agrees with the XPS results in Figure 1 and Table 1. In contrast, *I*(D)/*I*(D′) ≃ 6 for Ar-treated graphene, which indicated that both sp^3^- and vacancy-type defects were present. The plasma treatments in this study donated oxygen and hydrogen on pristine graphene, while a hexagonal in-plane structure of graphene was maintained despite the donation of sp^3^- and vacancy-type defects. Therefore, the defect type was controlled by choosing gas species in the plasma treatment; graphane, Ar-treated graphene, and graphoxide had sp^3^-type defect, sp^3^- and vacancy-type defects, and vacancy-type defect, produced by H_2_, Ar, and O_2_ plasma treatments, respectively.

### 3.2 Electrical Conductivity

**Figure 4:**
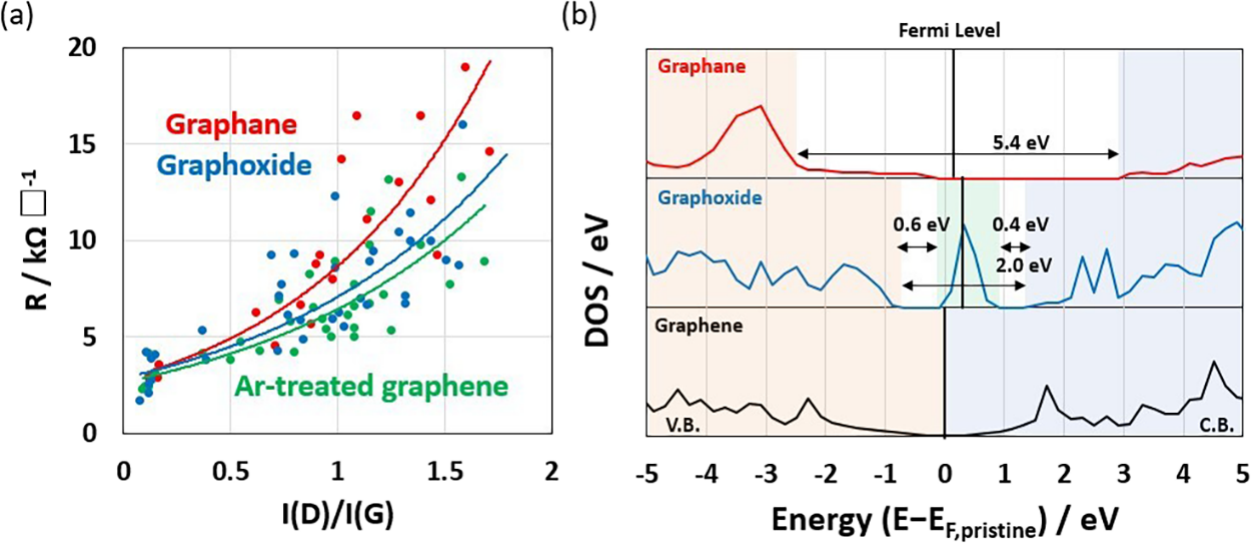
(a) Changes in sheet resistance with D/G intensity for different plasma treatments. (b) DOS of graphene, graphoxide, and graphane. Black bars represent the Fermi levels.

The sheet resistance of graphene (3.0 ± 0.6 kΩ □^–1^) increased linearly with treatment time by the different plasma treatments (Figure S8a). Graphane showed the largest increase in sheet resistance, which reached 15 kΩ □^–1^ after a treatment time of 20 s. In contrast, graphoxide and Ar-treated graphene showed smaller increases in the sheet resistance to 10 kΩ □^–1^. The large sheet resistance for graphane can be attributed to the increase in the D band intensity (Figure 3a). Even though the D band intensity of graphane did not increase beyond a treatment time of 10 s (Figure S6), the sheet resistance increased monotonically with the treatment time. Figure 4a plots the relationship between the sheet resistance and D band intensity. The sheet resistance increased linearly with the D band intensity when *I*(D)/*I*(G) < 1.0 but increased abruptly when *I*(D)/*I*(G) > 1.0. The D band intensity hardly increased when the graphene structure was mostly disordered. Lucchese et al. showed that the D/G intensity ratio decreased when the disordered regions were increasingly widespread, or in other words, the average distance between defects was shorter than 3 nm by increasing defect numbers.^42^ Graphane had a higher sheet resistance than graphoxide and Ar-treated graphene at all treatment times, because hydrogenation of graphene produces sp^3^ carbon more efficiently than the other plasma treatments (Figure 1 and Table 1), which weakens the electrical conduction path. For graphoxide and Ar-treated graphene, the creation of vacancy-type defects gradually weakened the electrical conduction path.^41^Figure 4b shows the density of states (DOS) of pristine graphene, graphoxide, and graphane (see Figure S7 for the corresponding structural models). Ideal graphene has a zero bandgap and becomes electric conductor, but actual graphene has a small sheet resistance (∼3 kΩ □^–1^ in this study), caused by the boundaries between graphene and defects. The actual bandgap energy of the pristine graphene was evaluated by the temperature-dependent sheet resistance (Figure S8b) and the bandgap energy was 0.01 eV, which was agreed with the ideal graphene in Figure 4b. Graphane and graphoxide had bandgaps of 5.4 and 2.0 eV, respectively, owing to hydrogenation and the insertion of hydroxyl groups. For graphoxide, the oxygen functional groups inserted a new intermediate band between the valence and conduction bands, which divided the original bandgap of 2.0 eV into smaller bandgaps of 0.6 and 0.4 eV. Two routes of the electron flow between the valence and conduction bands are proposed; the electron flow from the valence band of graphene (the orange region in Figure 4b) to the conduction band of graphene (the blue region) and the electron flow via the intermediate band (the green region) attributed to carbon atoms close to an oxygen functional group, which reduced the apparent bandgap to 0.6 eV. The trends for the bandgaps agreed with the experimental values for the sheet resistance, and the graphene-based materials all showed values typical of semiconductors.^43^

### 3.3 NH_3_ Gas-Sensing Performance

**Figure 5:**
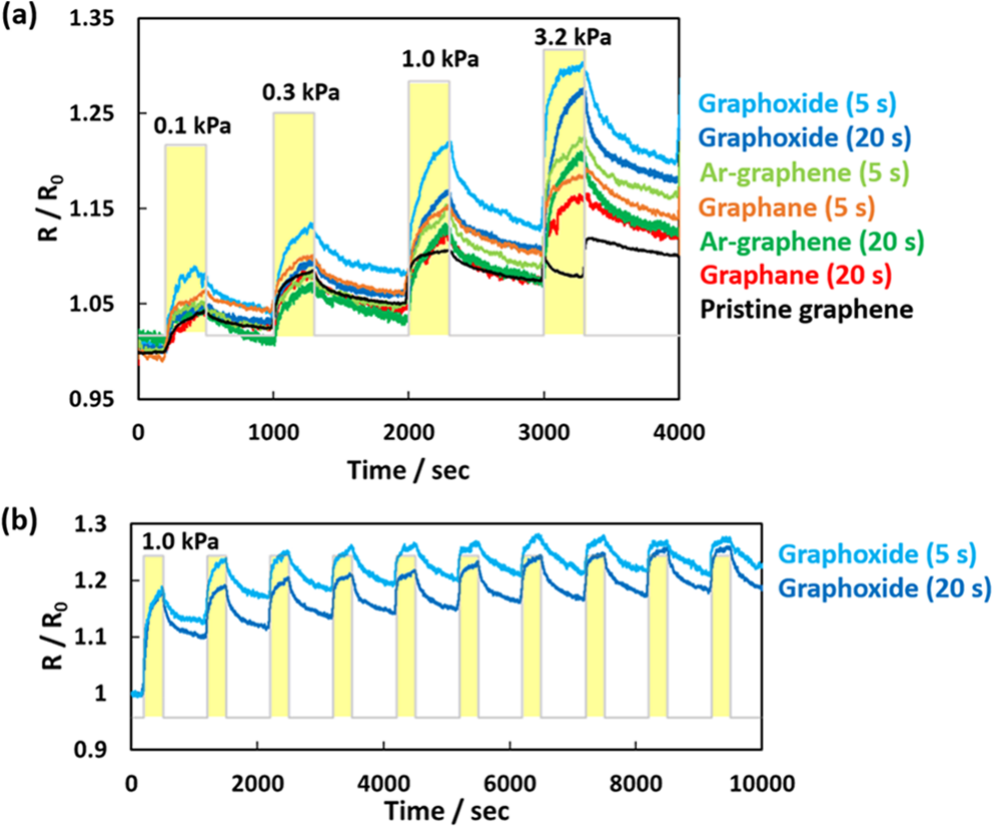
(a) Responses of pristine graphene, graphoxide, graphane, and Ar-treated graphene to NH_3_ gas pressures of 0.1–3.2 kPa. (b) Cyclability of sheet resistance of graphoxide prepared by treatment times of 5 and 20 s between NH_3_ gas pressures of in vacuo and 1.0 kPa.

Figure 5a shows the NH_3_ gas-sensing performances of pristine graphene, graphane, graphoxide, and Ar-treated graphene at 273 K. The sheet resistance increased proportionally with the NH_3_ gas pressure.^21,44,45^ For the pristine graphene, the sheet resistance increased slightly with NH_3_ gas injection, which agrees with our previous results.^24^ The change in sheet resistance was attributed to chemisorption of NH_3_ gas on the oxygen functional groups at the edges, although the amount of adsorption was hardly observed.^46^ Ricciardella and co-workers similarly reported that exfoliated graphene with a smooth surface and many defects demonstrated a quick and large response for NO_2_ gas.^47^ The change in sheet resistance of pristine graphene reached approximately 10% at a NH_3_ gas pressure of 1.0 kPa. The resistance changed only slightly at a gas pressure of 3.2 kPa. NH_3_ molecules might compete to be chemisorbed on a few active sites such as oxygen functional groups and then less affect the resistivity. Surprisingly, the sheet resistance increased during the evacuation process, which was observed only on the pristine graphene with repeatability (see the resistances in 3300 s in Figure 5a). Although we first anticipated that graphene structure was broken, the resistivity of pristine graphene was recovered after heating in vacuo. Those results supported the above competition mechanism of NH_3_ gas approaching to a few active sites and/or adsorbed NH_3_ molecules bridged a gap between graphene units, inducing the depression of the resistance. However, further analyses are needed to clarify the mechanism on pristine graphene. Functionalization by plasma treatment increased the sensitivity of graphene to NH_3_ gas. Graphoxide showed the highest sensitivity, followed by Ar-treated graphene and graphane. Graphoxide showed the largest changes in sheet resistance, which reached 30 and 27% at a NH_3_ gas pressure of 3.2 kPa with treatment times of 5 and 20 s, respectively. Although the changes in sheet resistance were mainly caused by NH_3_ physical adsorption and chemisorption on oxygen functional groups, those changes were larger with a treatment time of 5 s, which resulted in fewer oxygen functional groups (O/C = 0.12), than with a treatment time of 20 s, which resulted in more oxygen functional groups (O/C = 0.17). These results indicated that oxygen sparsely attached to graphene with a treatment time of 5 s, but electron conduction pass was inhibited by further oxidation close to oxidized carbons, which reduced the sensitivity to NH_3_ gas. Graphane sensed NH_3_ gas by weak hydrogen bonding. The change in sheet resistance was 15–17% at a NH_3_ gas pressure of 3.2 kPa, which indicates a lower sensitivity than graphoxide, but higher sensitivity than pristine graphene. Ar-treated graphene demonstrated a surprisingly high NH_3_ gas sensitivity with a change in sheet resistance of 20–22% at a NH_3_ gas pressure of 3.2 kPa. Ar-treated graphene had similar sp^3^ carbons and oxygen functional groups as graphane, but the additional presence of vacancy-type defects increased its NH_3_ gas sensitivity.

Figure 5b shows the cycle characteristics of the NH_3_ gas-sensing performance by graphoxide between NH_3_ gas pressures of in vacuo and 1.0 kPa for up to 10 cycles. All graphene-based materials demonstrated cyclability of the NH_3_ gas-sensing performance although the sheet resistances did not recover perfectly during only evacuation, especially after the first NH_3_ gas insertion by chemisorption. However, the obvious resistivity changes were continuously observed. The reversible and irreversible changes to sheet resistance were attributed to physical adsorption and chemisorption, respectively. Chemisorption induced large changes in sheet resistance, while it degraded the cyclability. However, the resistivity was perfectly recovered after heat treatment at 360 K for 3 h in a vacuum. Indeed, the resistance change by NH_3_ gas insertion at 330 K was recovered the initial resistance after only evacuation, although the sensing performance was decreased to about half resistance of that at 273 and 300 K.^24^ A treatment time of 20 s provided more physical adsorption sites than a treatment time of 5 s, which improved the cyclability. Similar trends were observed for graphane and Ar-treated graphene (Figure S9). The longer plasma treatments induced the oxidations of graphene, which was estimated from the 1 eV C 1s binding energy shift from 5 s-plasma-treated graphene to 20 s-plasma-treated graphene in Figures 1 and S4. However, the hydroxyl groups with less oxidative state strongly adsorbed NH_3_ and irreversible adsorption with chemisorption was thus observed in the short plasma-treated graphene.^19^ Those results indicated that NH_3_ molecules were preferentially adsorbed on the oxygen functional groups, especially on the hydroxy groups, the bandgap was then assumed to be increased by the change of the intermediate band in Figure 4b, and the sheet resistance was finally considerably increased, as shown in Figure 5. Table 2 shows the NH_3_ gas sensitivity in this work, and the previous works using graphene, graphene oxides, and reduced graphene oxides. The sensitivity of NH_3_ gas was mostly 3–30% on graphene and the related materials. The defective structures of graphene especially play important role in sensing NH_3_ gas.

**Table 2:** NH_3_ Gas Sensitivity in This Work and Previous Works^a^

materials	*S*/%	*C*/ppm	*T*	reference	materials	*S*/%	*C*/ppm	*T*	reference
graphene	5–10	1000–32 000	0 °C	in this work	graphene/mica	1–7	20–100	r.t.	Aziza et al.^45^
graphoxide	5–30	1000–32 000	0 °C	in this work	3D graphene foam	5–30	20–1,000	r.t.	Yavari et al.^44^
Ar-graphene	5–22	1000–32 000	0 °C	in this work	RGO	45	200	r.t.	Kehayias et al.^48^
graphane	5–17	1000–32 000	0 °C	in this work	RGO	2–5	100–1,000	r.t.	Minitha et al.^20^
graphene	3–30	75–400	70 °C	Wu et al.^49^	holey graphene oxide	3–11	1–50	r.t.	Yang et al.^21^
single-layer graphene	4–13	100–800	r.t.	Liang et al.^50^	RGO	2–22	5–40 with humidity	r.t.	Duy et al.^51^
graphene	3–24	1000	r.t.	Song et al.^52^	RGO	2–5	50	r.t.	Li et al.^53^
multilayer graphene	3–13	100–100 000	0 °C	Kitayama et al.^24^	3D SiO_2_/RGO	4–30	5–100	r.t.	Huang et al.^54^
F-graphene	–5–10	100–10 000	r.t.	Katkov et al.^55^	RGO	0–22	0–100	r.t.	Wang et al.^56^
P-doped graphene	5	100	r.t.	Niu et al.^57^	MXene/RGO	4–7	10–50	r.t.	Lee et al.^58^
S-/N-doped graphene	0–10	0–100	r.t.	Gavgani et al.^59^					

a Note: *S* and *C* represent the sensitivity of NH_3_ gas and the NH_3_ concentration.

## 4 Conclusions

In this study, graphene was functionalized by plasma treatment in O_2_, H_2_, and Ar environments. Graphoxide showed large increases in oxygen functional groups (12–17%) and sp^3^ carbons (13–18%) compared with pristine graphene (9 and 10%, respectively). Graphane showed a large increase in sp^3^ carbons (11–18%) and smaller increase in oxygen functional groups (11–13%). Ar-treated graphene was expected to show a decrease in oxygen functional groups but instead showed increases in both sp^3^ carbon and oxygen functional groups that were similar to those of graphane. The increase in oxygen functional groups for graphane and Ar-treated graphene was attributed to the respective plasma treatments producing dangling bonds that then reacted with O_2_ and water vapor after exposure in air. The Raman scattering spectra suggested that sp^3^-type defects were dominant for graphane, vacancy-type defects were dominant for graphoxide, and both sp^3^- and vacancy-type defects were present on Ar-treated graphene. The sheet resistance was correlated with the number of defects. At the same D/G intensity ratio, graphane had the highest sheet resistance followed by graphoxide, Ar-treated graphene, and pristine graphene. The DFT calculations indicated that graphane and graphoxide had bandgaps of 5.4 and 2.0 eV, respectively, even though ideal graphene has no bandgap. Here, graphoxide had an intermediate band from a CO group. The increased sheet resistances of graphane and graphoxide were attributed to the increased bandgaps. In contrast to the sheet resistance results, graphoxide showed the best NH_3_ gas-sensing performance, followed by Ar-treated graphene, graphane, and pristine graphene. This indicates that the vacancy-type defects of oxygen functional groups interact with NH_3_ gas more strongly than sp^3^-type defects. Short treatment times generated highly active sites inducing NH_3_ chemisorption, whereas long treatment times also generated fewer active sites inducing NH_3_ physical adsorption, which improved the cyclability of the NH_3_ gas-sensing performance. The results indicated that functionalizing graphene by oxidation and hydrogenation generates noble materials with a superior NH_3_ gas-sensing performance. The further study on the properties of various gas sensing is necessary to evaluate the performances on graphene-like materials.

## Supporting Information Available

Procedure of graphene preparation and gas-sensing tests; XPS of graphene family; relationship between oxygen groups and sp^3^ carbon; Raman scattering spectra of functionalized graphene; sheet resistances; and structural models of graphene family (PDF)
