# Supplementary material for: Graphene Functionalization by O2, H2, and Ar Plasma Treatments for Improved NH3 Gas Sensing
Source: ACS Appl Mater Interfaces. 2024 Dec 26;17(1):1992–9. doi: 10.1021/acsami.4c17257 (PMC11783355; doi:10.1021/acsami.4c17257)
Supplement: Supplementary file 1 — am4c17257_si_001.pdf [file am4c17257_si_001.pdf]

Supporting Information

Graphene functionalization by O_2, H_2, and Ar plasma treatments for improved NH_3 gas sensing

Sogo Iwakami,[†] Shunya Yakushiji,[†] and Tomonori Ohba[†,*]

[†] Graduate school of Science, Chiba University, 1-33 Yayoi, Inage, Chiba 263-8522, Japan

[*] *Corresponding author*. E-mail address: ohba@chiba-u.jp (T. Ohba)

Table S1. Ratios of sp^2 carbons, sp^3 carbons, and oxygen functional groups of pristine graphene and functionalized graphene.

	sp^2 carbon	sp^3 carbon	Oxygen functional groups
Graphene	80.9% ± 1.3%	10.4% ± 1.4%	8.7% ± 0.2%
Graphoxide	74.8% ± 1.1%	13.1% ± 0.6%	12.1% ± 0.6%
Graphane	77.7% ± 1.4%	11.4% ± 1.0%	10.8% ± 0.4%
Ar-etched graphene	76.2% ± 1.2%	13.2% ± 0.1%	10.7% ± 1.1%

Note: Functionalized graphene types were treated for 5 s. Values were obtained by calculating the areas under the peaks in the XPS C1s spectra.

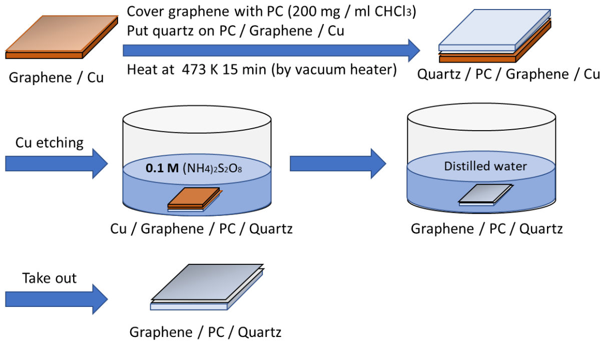

Figure S1. Graphene preparation procedure.

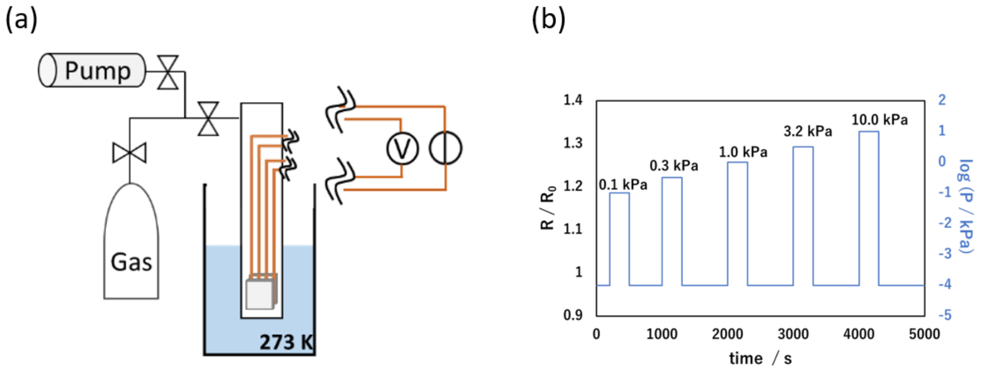

Figure S2. Gas-sensing performance measurement: (a) system setup and (b) measurement conditions.

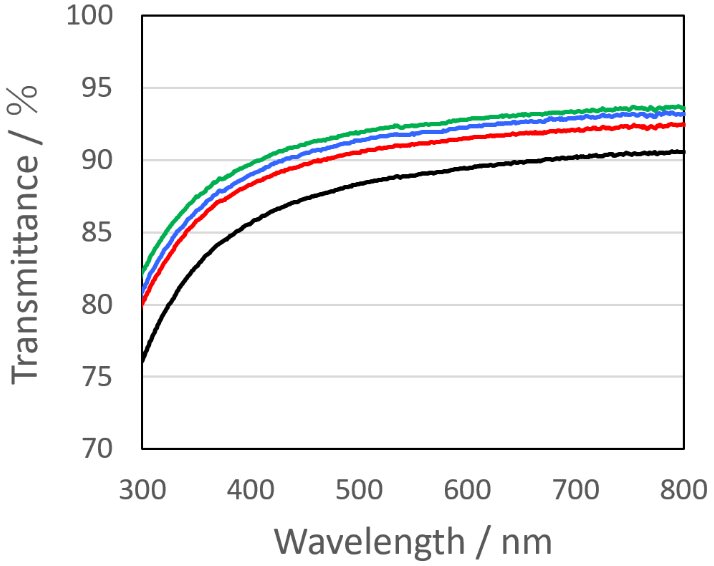

Figure S3. UV-VIS spectra of pristine graphene (black), graphoxide (blue), graphane (red), and Ar-treated graphene (green).

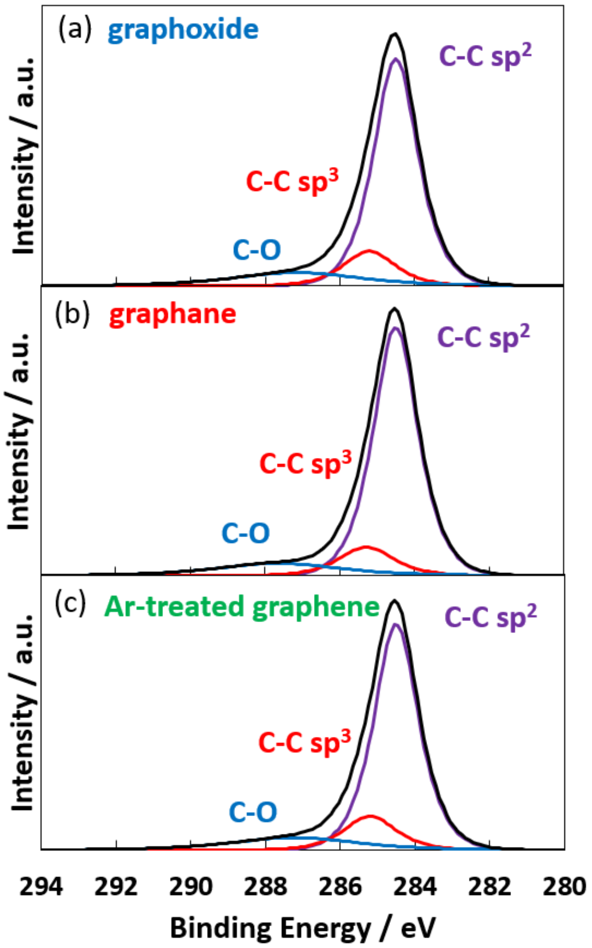

Figure S4. XPS C1s spectra of different functionalized graphene types at a treatment time of 5 s: (a) graphoxide, (b) graphane, and (c) Ar-treated graphene.

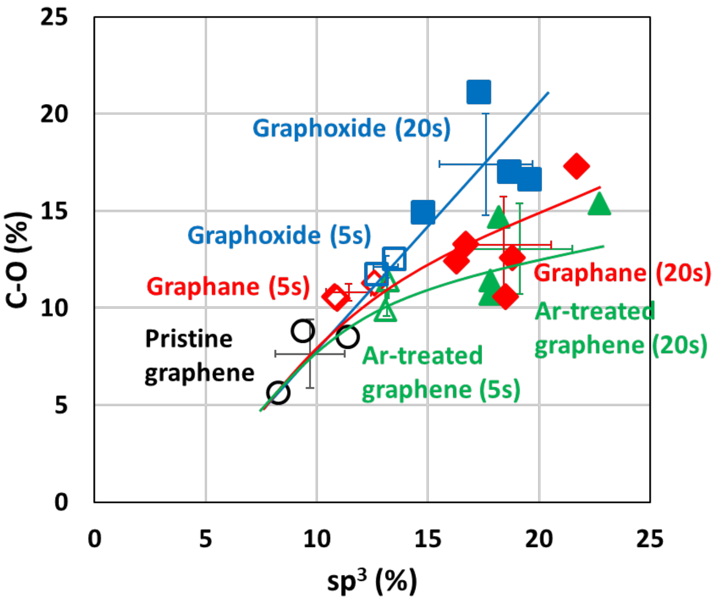

Figure S5. Relationship between oxygen functional groups and sp^3 carbon obtained from peaks in XPS C1s spectra.

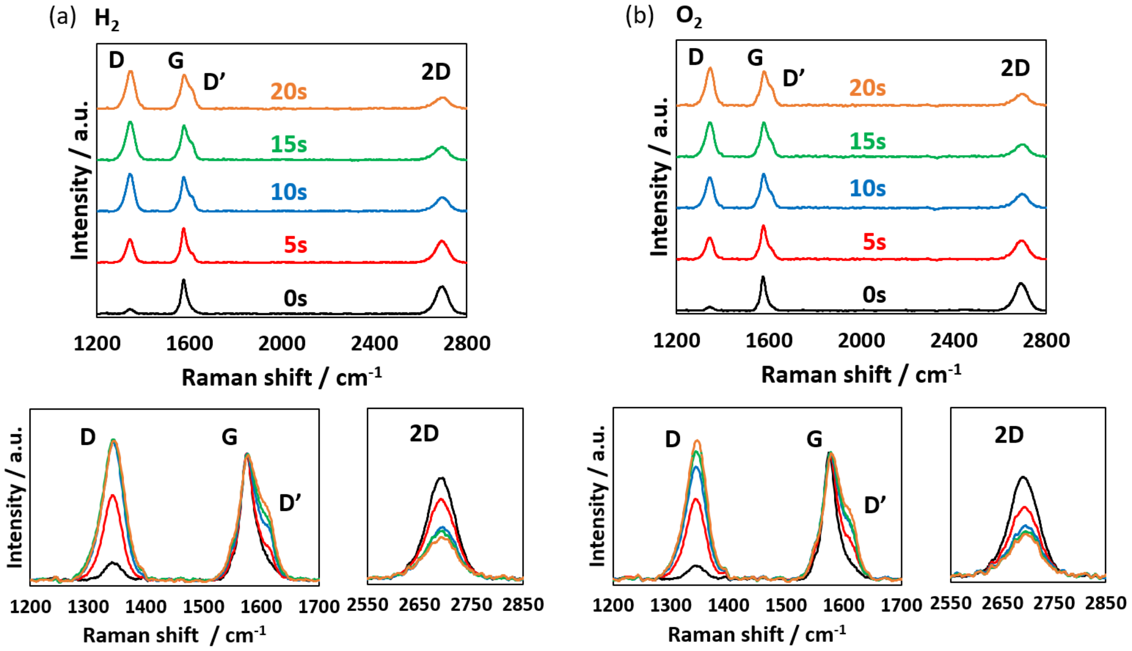

Figure S6. Raman scattering spectra of functionalized graphene with different treatment times: (a) graphane (b) graphoxide.

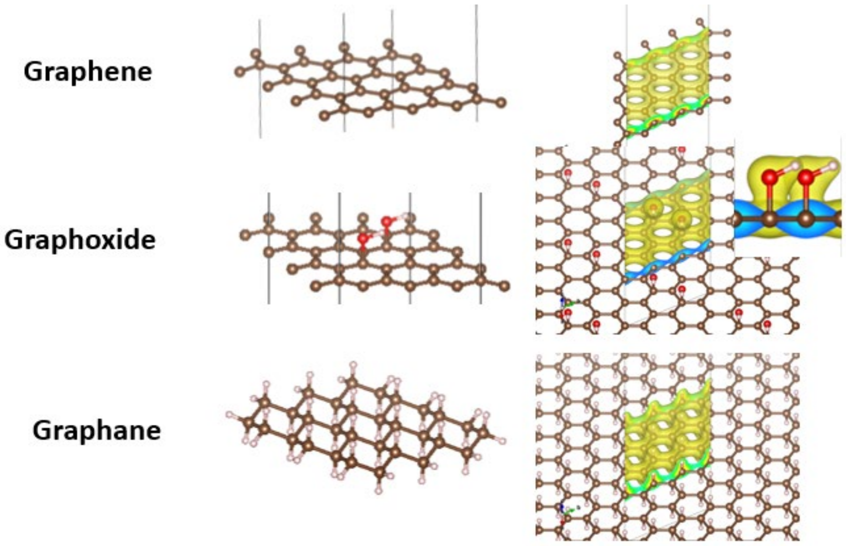

Figure S7. Structural models of pristine graphene, graphoxide, and graphane (left), and isosurfaces (right).

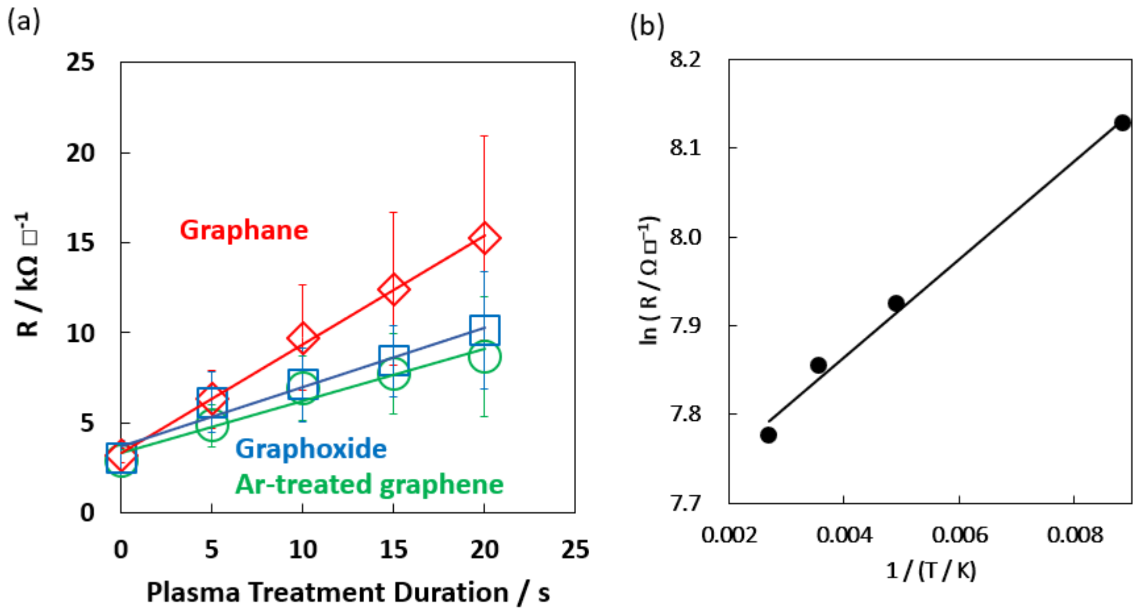

Figure S8. (a) Changes in sheet resistance with treatment time for graphoxide (squares), graphane (diamonds), and Ar-treated graphene (circles). (b) The sheet resistance changes as function of temperature.

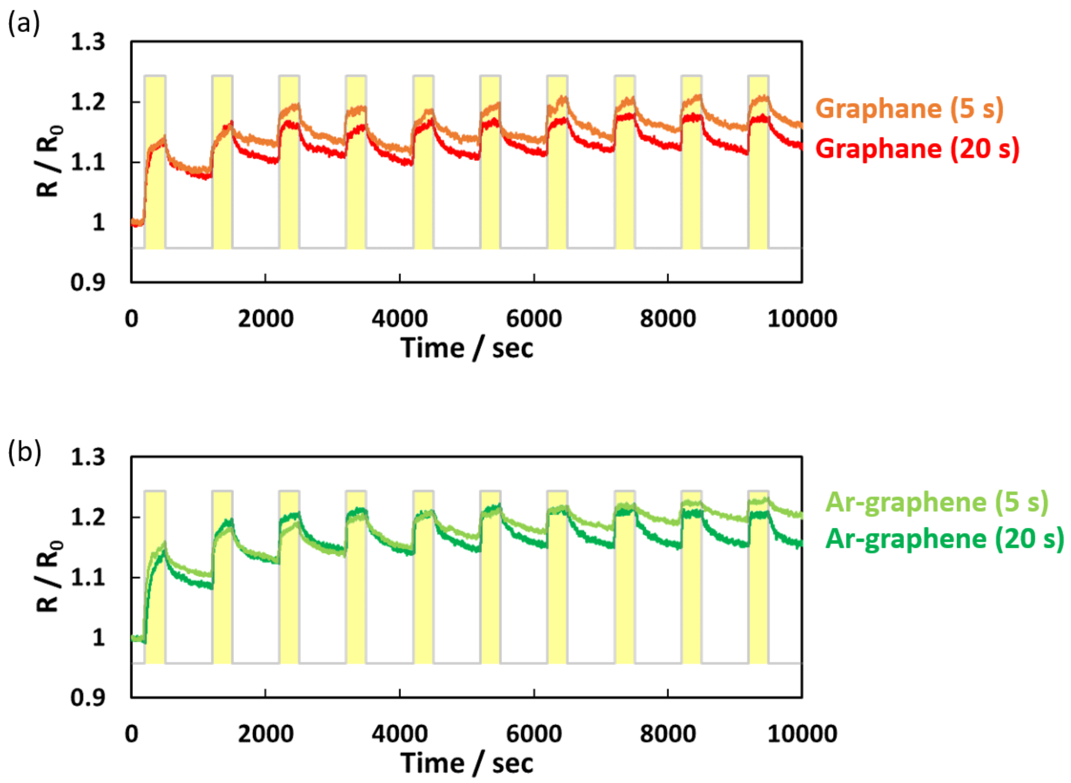

Figure S9. Cyclability of sheet resistance between NH_3 gas pressures of in vacuo and 0.1 kPa: (a) graphane and (b) Ar-treated graphene with treatment times of 5 and 20 s.
